# The association between price, competition, and demand factors on private sector anti-malarial stocking and sales in western Kenya: considerations for the AMFm subsidy

**DOI:** 10.1186/1475-2875-12-186

**Published:** 2013-06-05

**Authors:** Wendy Prudhomme O’Meara, Andrew Obala, Harsha Thirumurthy, Barasa Khwa-Otsyula

**Affiliations:** 1 Division of Infectious Diseases, Duke University School of Medicine, Durham, NC, USA; 2 Duke Global Health Institute, Durham, NC, USA; 3 College of Health Sciences, Moi University School of Public Health, Eldoret, Kenya; 4 Webuye Demographic Surveillance Site Scientific Steering Committee, Eldoret, Kenya; 5 College of Health Sciences, Moi University School of Medicine, Eldoret, Kenya; 6 School of Public Health, University of North Carolina, Chapel Hill, USA

**Keywords:** Malaria, Access, Subsidized medicines, Retail

## Abstract

**Background:**

Households in sub-Saharan Africa are highly reliant on the retail sector for obtaining treatment for malaria fevers and other illnesses. As donors and governments seek to promote the use of artemisinin combination therapy in malaria-endemic areas through subsidized anti-malarials offered in the retail sector, understanding the stocking and pricing decisions of retail outlets is vital.

**Methods:**

A survey of all medicine retailers serving Bungoma East District in western Kenya was conducted three months after the launch of the AMFm subsidy in Kenya. The survey obtained information on each anti-malarial in stock: brand name, price, sales volume, outlet characteristics and GPS co-ordinates. These data were matched to household-level data from the Webuye Health and Demographic Surveillance System, from which population density and fever prevalence near each shop were determined. Regression analysis was used to identify the factors associated with retailers’ likelihood of stocking subsidized artemether lumefantrine (AL) and the association between price and sales for AL, quinine and sulphadoxine-pyrimethamine (SP).

**Results:**

Ninety-seven retail outlets in the study area were surveyed; 11% of outlets stocked subsidized AL. Size of the outlet and having a pharmacist on staff were associated with greater likelihood of stocking subsidized AL. In the multivariable model, total volume of anti-malarial sales was associated with greater likelihood of stocking subsidized AL and competition was important; likelihood of stocking subsidized AL was considerably higher if the nearest neighbour stocked subsidized AL. Price was a significant predictor of sales volume for all three types of anti-malarials but the relationship varied, with the largest price sensitivity found for SP drugs.

**Conclusion:**

The results suggest that helping small outlets overcome the constraints to stocking subsidized AL should be a priority. Competition between retailers and prices can play an important role in greater adoption of AL.

## Background

In sub-Saharan Africa, retail shops, pharmacies and chemists are important sources of treatment for fevers, including those related to malaria [1-3]. In Kenya, 17 to 83% of fevers are first treated with medicines purchased from shops [4]. Even when treatment is eventually sought in the formal health sector, a retail outlet may be the first source of treatment. Moreover, when drugs are not available in government health facilities, patients are generally referred to the retail sector to purchase the prescribed treatment. Patients may even bypass public facilities altogether when stock outs are common or known.

The retail market of anti-malarials is a complex landscape consisting of many different wholesale-to-retail pathways with variable numbers of links; different types of outlets ranging from registered pharmacies to general stores; dozens of different types and brands of anti-malarials, and widely varying prices [5-8]. Within this diversity of choice, customer preferences and demand can have a strong influence on shop owners’ decisions regarding which drugs to stock [7,9]. Because price is a crucial factor in patient demand, particularly in resource-limited settings, many patients seeking treatment in the retail sector purchase inexpensive, but ineffective, anti-malarials rather than more expensive artemisinin combination therapy (ACT) that the World Health Organization (WHO) has recommended for treatment of uncomplicated malaria [2,10-12].

In order to increase availability of low-cost ACT in the retail sector, the Affordable Medicine Facility malaria (AMFm) was introduced on a pilot basis in 2010 in eight countries [13]. The AMFm provides high quality ACT to wholesalers at heavily subsidized prices. Although the programme has suggested retail prices for AMFm products, the retail price of the subsidized ACT is not regulated; wholesalers and retailers are free to establish prices as they would with any commodity. Only the first-line buyers (first wholesaler) in the pilot countries have signed price agreements to pass down subsidies. The effectiveness of AMFm from a public health standpoint depends on the savings being passed down to the retailer and ultimately to the customer. An important consideration in this regard is the complex nature of the retail sector, which includes a large number of small-scale outlets, a requirement for profitability, and a different client dynamic than is found in the formal health sector. So far there is little information in the literature describing what factors influence anti-malarial stocking and pricing in the retail sector.

In order to understand the potential impact of AMFm as well as the results of the AMFm pilot, a better understanding of the reasons why retailers choose to stock specific drugs and what determines the volume of different types of drugs sold in these outlets is required. Moreover, the sensitivity of demand to the price of drugs is an important factor in determining the effectiveness of the subsidy. If volume sold is unrelated to price, this would suggest that a subsidy alone will have less of an effect on consumer demand than other factors, for example branding or consumer awareness.

In Kenya, the recommended ACT is artemether-lumefantrine (AL) and the AMFm provides subsidized AL at about 10 Kenyan shillings (KES, US$0.12) per course with a target price to the end user of 40 KES (US$0.50) per treatment course. A survey of all medicine retailers in a district in western Kenya was conducted shortly after the nationwide launch of AMFm. Characteristics of surveyed retail outlets are described and factors associated with AL stocking decisions and sales of different types of anti-malarials are examined.

## Methods

### Study area

This study was conducted in the Webuye Health and Demographic Surveillance System (HDSS) of Bungoma East District, western Kenya. The HDSS is located in Webuye Division. Webuye Town is approximately 80 km west of Eldoret and 50 km east of Kenya-Uganda border. The Nairobi-Uganda highway bisects the larger Bungoma County, thereby opening up the county for numerous economic activities. However, most households are poor, with 52% of the population living below the poverty line, and cannot access social amenities such as water and electricity. The poverty line is defined as those families living on less than 1,562 KES (~20USD) per month. This is the minimum requirement for essential food and non-food items as defined by the Kenya National Bureau of Statistics, Kenya Integrated Household Budget Survey 2005/06. The area has a tropical climate and lies at an elevation of 1,523 m above sea level. It experiences an average annual temperature of 24°C, and receives high rainfall ranging from 1,200 to 1,800 mm annually [14]. The main economic activities include sugarcane farming as a cash crop, while maize, sorghum and millet are cultivated as subsistence crops. Dairy and poultry farming are also widely practiced. The largest paper factory in Africa and chemical processors are located within Webuye Town [15]. The HDSS is described in detail elsewhere [16]. Malaria burden in Bungoma East is high, due to the suitable climate and elevation, coupled with low coverage of insecticide-treated bed nets [17,18].

### Data collection

A survey of all medicine retailers serving the HDSS was undertaken in November 2010. Details of the survey are reported elsewhere [7]. Briefly, a complete census of retail medicine outlets that sell primarily human medicines (excluding general stores) stocking malaria medicines was carried out in August 2010. Outlets were included regardless of legal registration status. Outlets that were within 5 km of the HDSS border were included in order to capture all outlets that may serve HDSS residents. Interviews were conducted with the primary shop attendant in November 2010. The survey was carried out a few months after the launch of the AMFm subsidy in August 2010. Interviewers administered the questionnaire and took a complete inventory of anti-malarials in stock on the day of the survey. The survey also recorded the price for a treatment course of each drug, brand name, active ingredients, and number of treatment courses sold in the past one week. GPS co-ordinates of each retailer were also recorded.

### Geographic variables

Household GPS co-ordinates were used to describe the population density and fever prevalence around outlets that were located within the HDSS. Household co-ordinates were used to make a density raster using a kernel density function with a radius of 0.5 km. The value of the raster at the location of each outlet describes the population density within a 0.5 km radius around each shop. The same procedure was repeated for households with fevers according to the most recent HDSS household survey. The fever density variable describes the burden of fevers within the vicinity of each retailer. The ratio of fever density to household density was also calculated for each retailer to estimate the period prevalence of fevers around each shop. These three variables – population density, fever density and prevalence in the vicinity of each shop – describe the potential for local demand. These variables were only calculated for retail outlets located entirely within the HDSS. Household characteristics – acres of land owned and whether a family grows cash crops – were also used to make density rasters and values were extracted to outlet locations. The percent of households growing cash crops, level of education of the household head, and average acres of land owned by households around each outlet were used as proxies for wealth amongst the households closest to each. Density rasters and distance between neighbouring outlets and health facilities was calculated in ArcMap 10 (ESRI).

### Data analysis

The analyses investigated three major shop-level outcome variables: the probability of stocking AL, the probability of stocking subsidized AL from the AMFm programme, and the volume of three different types of anti-malarials sold over the last week [AL, quinine, and sulphadoxine-pyrimethamine (SP)]. The log of drug sales volume was taken after adding one so that zero values could be included in the analysis. The association between these outcome variables and the following factors was examined: outlet size, quality, presence of nearby competitors and population characteristics. Price of the relevant drug was included when the outcome variable was sales volume of anti-malarials. The overall size of the retail outlet was measured by the total number of staff and the total volume of all anti-malarials sold in the last week. Quality was measured by the number of pharmacists and nurses on staff and whether untrained staff dispensed medications. The number of other medicine retailers selling anti-malarials within 1 km was used as a proxy for competition. Presence of a government facility within 1 km and whether the nearest competitor sold subsidized AL were included in the analysis. Population characteristics for each outlet included household density, fever density and prevalence, density of cash crop farming, acres of land owned by households, and household head’s education.

To estimate the relationship between price and volume sold for each drug type, a regression model of the log of sales volume on the log of the price was used, while controlling for other factors that influence demand such as the local disease burden and population density. Chi-square tests and t-tests were used to explore the relationship between independent variables and retail outlet characteristics. Multivariate normal and logistic regression models were estimated to determine factors that influence retailer’s decision to stock AL and subsidized AL and factors that influence sales volume of the most commonly stocked anti-malarials. P-values <0.10 were considered significant due to the small number of outlets in the analysis. Analysis was done using Stata 11 (StataCorp).

### Ethical approval

The study was approved by the Moi University Institutional Research and Ethics Committee and the Duke University Institutional Review Board. Consent from local community leaders and the Webuye Health and Demographic Surveillance Site Scientific Steering Committee was also sought and granted.

## Results

### Characteristics of medicine retail outlets stocking anti-malarials

Ninety-seven outlets that regularly stocked and sold anti-malarials were surveyed and included in the analysis (Table 1). The median number of different brands of anti-malarials per outlet was two (range 1–8), with 28 stocking a single brand of anti-malarial.

**Table 1:** Characteristics of surveyed outlets

**Shop characteristics (n = 97)**		
*Size*		
Mean volume sold per shop (SD)	19.7	(27.6)
Median number of drug brands per shop (range)	2	(1–8)
Median number of personnel (range)	1	(1–6)
*Quality*		
Percent with a pharmacist or pharmaceutical technologist	46.4	
Percent with a nurse or nurse aide	39.2	
Percent with untrained personnel dispensing	25	
*Competition*		
Neighbours within 1 km (median, range)	2	(0–12)
Percent of shops within 1 km of government facility	42.6	
*Demand (n = 33)*		
Mean HH density around each shop (SD)	108	(80)
Mean fever density around each shop (SD)	22.8	(16.9)
Fever prevalence in households around each shop (SD)	0.23	(0.12)
Proportion of households with cash crop farming around each shop	25.1	(21.1)
Acres of land owned (average in HDSS)	1.8	(3.74)
Head education (all HDSS)		
*Primary or below*	60.5%	
*Secondary*	30.9%	
*Above secondary*	8.6%	

Supply-side factors collected through the survey include size of the outlet, training of the staff, and competition. The median number of neighbours within 1 km was two, but ranged from zero to 12. A government facility was located within 1 km for 42.6% of the outlets.

The number of staff ranged from one to six and 62% of retail outlets had only one staff member. On average, shops sold ~20 courses of anti-malarials in the preceding one-week period; 46.4% of shops had a nurse or nurse-aide on staff and 39.2% had a pharmacist or pharmaceutical technologist; 25% of shops had untrained staff (no relevant training or education) dispensing drugs.

Demand-side factors including local population density and fever burden were estimated for shops within the HDSS (n = 33) by extracting local population characteristics around each outlet. The mean density of households (number per 0.8 sq km) around an outlet was 108 and the mean number of fevers within these households in the last six months was 22.8. The ratio of fevers to households (local period prevalence per 0.8 sq km) was 0.23.

Average household characteristics that were predicted to reflect purchasing power (acres of land owned, education of the head and cash crop farming) were also estimated around each outlet. On average, 23% of households within 0.8 sq km of outlets were cash crop farmers and households owned on average 1.8 acres of land. Overall, 39% of household heads had secondary education or above.

**Figure 1:**
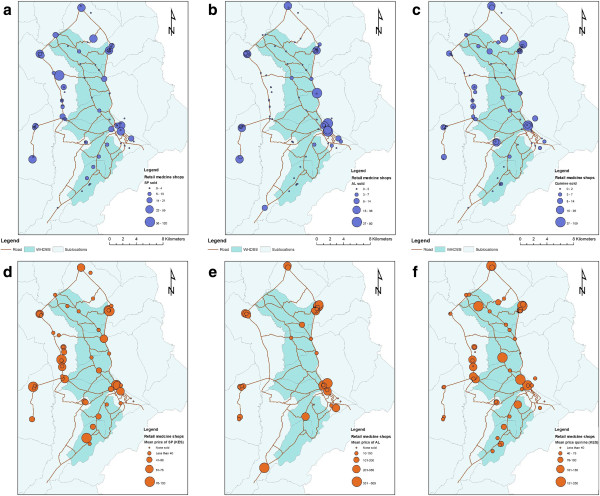
**Map of study area showing sales volume and price of antimalarials in retail outlets of Bungoma East district. a**, **b**, **c**. Sales volume in the last one week for sulphadoxine-pyrimethamine, artemether lumefantrine and quinine, respectively. **d**, **e**, **f**. Price of sulphadoxine-pyrimethamine, artemether lumefantrine, and quinine, respectively. The size of the circle scales with the magnitude of the variable depicted.

The average price of a treatment course of AL was 2.73 USD and ranged from 0.38 to 7.50 USD. The average price for quinine (1.21 USD, range 0.25-5.63 USD) and SP (0.65 USD, range 0.19-1.50 USD) was lower than for AL. The geographic availability of SP and quinine is much greater than for AL (Figure 1). Pockets of AL availability coincide with peri-urban areas and market centres where price heterogeneity is greater. However, lower prices for AL are found in areas where shops are sparser.

### Anti-malarial stocking practices

Some 22% of outlets stock any brand of AL, with a total of 13 different brands observed. Table 2 reports unadjusted and adjusted odds ratios for the analysis of AL and AMFm AL stocking practices. There was statistically significant correlation between outlet characteristics and whether or not they carried any brand of AL. The number of other anti-malarial outlets in the vicinity increased the likelihood of stocking AL as did the size of the outlet, determined by both total staff and total anti-malarial doses sold in the last week. Outlets with a pharmacist on staff were far more likely to carry AL (OR = 4.92; p < 0.01) whereas those with a nurse on staff were less likely to carry AL (OR = 0.28; p < 0.01). Increasing numbers of households in the vicinity that farmed cash crops slightly reduced the odds of stocking AL (OR = 0.93, p = 0.024). In the multivariable model, the size of the outlet (OR = 3.56; p = 0.02) and nearby competition (OR = 1.13; p = 0.08) were correlated with carrying any AL. Shop attendant training (pharmacist, nurse or no training) was no longer statistically significant when the effect was corrected for size of the outlet.

**Table 2:** Stocking artemether-lumefantrine and subsidized artemether-lumefantrine

	**Variable**	**Stocking any AL**		**Stocking AMFm AL**	
		**Unadjusted OR (p)**	Adjusted OR (p)*	**Unadjusted OR (p)**	**Adjusted OR (p)**
*Size*	Number of staff	3.74 (p = 0.001)	3.56 (0.019)	1.92 (p = 0.049)	1.14 (0.78)
	Total volume sold in last week	1.05 (p = 0.001)	1.04 (0.015)	1.04 (p = 0.001)	1.03 (0.03)
*Quality*	Pharmacist on staff	4.92 (p = 0.000)	2.45 (0.215)	3.53 (p = 0.076)	1.49 (0.8)
	Nurse on staff	0.28 (p = 0.005)	0.5 (0.375)	0.31 (p = 0.15)	0.52 (0.68)
	Untrained staff dispensing medicine	0.76 (p = 0.57)	-	0.69 (p = 0.65)	0.28 (0.4)
*Competition*	Number of neighbours within 1 km	1.16 (p = 0.01)	1.13 (0.08)	1.05 (p = 0.6)	1.07 (0.52)
	Nearest govt. facility within 1 km	1.19 (p = 0.68)	0.47 (0.20)	2.65 (p = 0.14)	0.69 (0.69)
	Nearest neighbour has subsidized AL		-	7.13 (p = 0.005)	6.4 (0.09)
*Demand*	Household density	0.99 (p = 0.62)	-	-	
	Fever density	0.97 (0.25)	-	-	
	Fever prevalence in households	0.06 (p = 0.21)	-	-	
	Density of cash crop farming	0.93 (0.024)	-	-	
	Acres of land owned	0.99 (0.074)	-	-	
	Head education	0.99 (0.52)	-	-	

*non-geographic variables only were used for the multivariable regression because the number of outlets within the DSS (n = 33) was much smaller than the overall sample size.

Eleven percent of retail medicine outlets in the survey carried subsidized AL. In order to understand if the determinants of stocking any AL were different than those correlated with stocking subsidized AL, the analyses were repeated looking at whether an outlet stocked subsidized AL. Similar to the results for any AL, training and size of the outlet were important predictors of stocking subsidized AL. Having a pharmacist on staff was also correlated with stocking subsidized AL. In the multivariable model, total volume of anti-malarial sales remained significantly but weakly correlated with stocking subsidized AL (OR: 1.03; p = 0.03). Competition appears to play a large role in determining whether shops stock subsidized AL. Outlets whose nearest competitor carried subsidized AL were six times more likely to also carry subsidized AL (OR: 6.4; p = 0.09).

### Sales volume and price

In order to analyse factors associated with the volume of anti-malarials sold by an outlet, the log of volume sold was used as the outcome variable. Other factors considered were similar to the analyses of stocking practices, including competition, local household and fever density, outlet size and staff training. The analysis was repeated for the three most commonly stocked drugs – AL, SP and quinine (Table 3).

**Table 3:** Relationship between sales volume, price and outlet characteristics

**Theme**	**Variable**	**SP**		**AL**		**Quinine**	
		**Unadjusted effect size (p)**	**Adjusted effect size (p)**	**Unadjusted effect size (p)**	**Adjusted effect size (p)**	**Unadjusted effect size (p)**	**Adjusted effect size (p)**
*Price*	Log price	-0.78 (0.002)	-0.92 (0.000)	-0.59 (0.02)	-0.44 (0.19)	-0.34 (0.02)	-0.29 (0.15)
*Size*	Number of staff	0.24 (0.25)	0.43 (0.06)	0.12 (0.51)	0.27 (0.09)	0.09 (0.48)	0.11 (0.55)
*Quality*	Pharmacist on staff	-0.02 (0.95)	-1.15 (0.08)	0.40 (0.24)	1.06 (0.22)	0.36 (0.11)	0.15 (0.78)
	Nurse on staff	-0.49 (0.08)	-1.57 (0.02)	0.040 (0.92)	0.69 (0.48)	-0.24 (0.29)	-0.5 (0.92)
	Untrained staff dispensing medicine	0.67 (0.05)	-0.47 (0.32)	-0.13 (0.76)	0.16 (0.84)	-0.09 (0.73)	-0.19 (0.66)
*Competition*	Number of neighbours within 1 km	0.03 (0.46)	0.05 (0.22)	-0.06 (0.15)	-0.04 (0.33)	0.004 (0.88)	0.002 (0.96)
	Nearest govt. facility within 1 km	-0.02 (0.95)	-0.13 (0.64)	0.53 (0.14)	0.38 (0.21)	0.39 (0.08)	0.26 (0.37)
	Nearest neighbour has subsidized AL	0.5 (0.86)	0.29 (0.51)	-0.09 (0.34)	-1.09 (0.06)	0.31 (0.94)	0.04 (0.04)
*Demand*	Household density	0.01 (0.198)	-	0.002 (0.88)	-	-0.002 (0.68)	-
	Fever density	0.053 (0.089)	-	0.03 (0.59)	-	-0.007 (0.76)	-
	Fever prevalence in households	2.4 (0.59)	-	15.1 (0.14)	-	-0.51 (0.90)	-
	Density of cash crop farming	0.01 (0.76)		0.016 (0.77)		-0.03 (0.11)	
	Acres of land owned	0.002 (0.40)		0.01 (0.45)		-0.003 (0.33)	
	Head education	0.004 (0.18)		0.002 (0.83)		-0.001 (0.73)	
		*n = 26 for geographic variables*		*n = 11 for geographic variables*		*n = 30 for geographic variables*	

**Figure 2:**
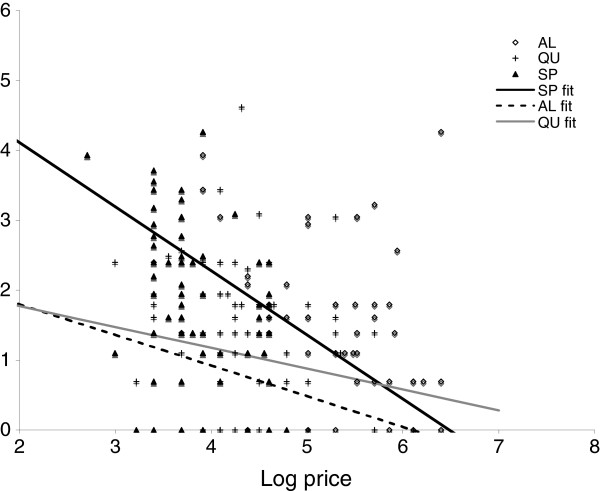
**Price *versus *sales volume for artemether lumefantrine, sulphadoxine-pyrimethamine and quinine.** Lines represent the slope of co-efficient of log price in the multivariate logistic regression models.

In the unadjusted analysis, price was a statistically significant predictor of sales volume for all three types of drugs – AL, SP and quinine – but the magnitude of the relationship varied by drug type (Figure 2). The strongest association between price and sales was found for SP and the weakest association was found for quinine. The negative co-efficient indicates that the volume of sales was inversely related to price, as expected. In the multivariable model for SP, 1% increase in the price of SP was associated with a 0.92% decrease in quantity sold (p < 0.01). For AL, on the other hand, a 1% increase in price was associated with a 0.44% decrease in quantity sold (p = 0.19).

Competition was an important determinant of sales volume for AL – the number of courses of AL sold declines significantly if the nearest neighbour had AL. Stores with more staff members sold more AL and SP, but stores with trained staff sold less SP even when correcting for size of the shop. Local household density was not correlated with sales volume for any drug in the univariate analyses. Fever density was correlated with SP sales (p = 0.089), but the result was marginally statistically significant.

## Discussion

Results from this study show that, shortly after the rollout of the AMFm subsidy programme, larger outlets with more staff, greater sales volume, and a pharmacist on staff were more likely to stock AL and subsidized AL. The presence of AL or subsidized AL in nearby outlets increased the odds of a shop selling AL and subsidized AL. Shops with neighbours selling AL also sold AL for lower prices. Overall, volume of anti-malarial sales was not as dependent on price as expected, particularly for AL.

The retail sector is an important source of treatment for fevers in malaria-endemic areas. Achieving the global target of 80% of childhood fevers treated with an appropriate anti-malarial within 48 hours of onset [1] will not be possible without involving the retail sector in many settings. Pilot studies of anti-malarial subsidies demonstrated significant improvements in the availability of ACT in the retail sector and the percent of fevers being treated with an effective anti-malarial [19,20]. In 2010, the AMFm subsidy was launched in eight countries to make effective ACT accessible and affordable in the retail sector. A previous report from Kenya showed that within just a few months of the launch in Kenya, subsidized ACT was available in retail shops in rural, malaria-endemic areas [7] and subsidized brands were markedly less expensive than other ACT. However, the price of AMFm-subsidized ACT still varied by nearly an order of magnitude between shops and in only one shop was AMFm-brand AL being offered at the recommended retail price. The price heterogeneity between different brands of the same drug, and even the same brands of the same drug, was also observed for outdated anti-malarials such as SP and quinine (Figure 1).

In a pilot study in Tanzania, 25% of retail outlets selling anti-malarials still did not stock ACT one year after implementation of the subsidy [20]. The factors that are correlated with stocking AL and subsidized AL shed light on the potential for success of antimalarial subsidies in the retail sector. The results in this paper are important for understanding how to improve uptake of subsidized drugs amongst retail outlets and understanding what other factors may increase the market share of subsidized drugs.

There was a consistently negative association between the price of drugs and shop-level sales of the drug. More importantly, the co-efficient estimated indicated that sales of anti-malarials were not extremely sensitive to price. This was especially true for quinine and AL and less so for SP. This was surprising given the wide range of prices observed for the same class of drug. This may be due to the fact that resource-poor households are driven to obtain anti-malarials when their children have a fever regardless of the price of these drugs (within a reasonable price range). They may be self-financing their purchases by reductions in consumption of other important items, such as food or transportation. The lack of correlation between the price of quinine and SP with doses sold challenges the assumption of the AMFm programme that the price of ACT is the most important barrier to increasing their market share. The AMFm subsidy will have to contend with the challenge of getting households to switch away from older medications. This is consistent with results from the AMFm evaluation, which showed that availability of ACT in retail shops was limited when implementation of supporting interventions such as community awareness campaigns was weak. On a country-wide scale, the availability of AMFm ACT in the retail sector did not seem to be related to the median price of the AMFm ACT [21]. Thus, price alone may not drive the success of the AMFm programme, as demand for specific anti-malarials may be dominated by other factors.

Few stores stocked AL or subsidized AL. Larger stores with more staff and larger sales volumes, probably corresponding to more rapid turnover of their inventory, are more likely to stock AL and subsidized AL. Larger stores may have more capital and can stock newer drugs or drugs that do not sell as quickly. The ineffective, outdated anti-malarial SP is sold in higher volumes from stores with untrained staff, even when correcting for the size of the store. Larger stores sell more quinine and AL, even when adjusting for price, but whether they have trained staff such as a nurse or pharmacist does not affect sales.

Similar to a study in Tanzania [22], the results presented here demonstrate that competition affects the likelihood of stocking subsidized AL. If the nearest competitors stock subsidized AL, it increases the likelihood of also stocking subsidized AL by six-fold. The strongest evidence for ascribing this to a competition effect comes from the added observation that, on average, an outlet sells less AL if the nearest neighbour has subsidized AL*.* However, this analysis is based on cross-sectional data and the availability of subsidized AL in individual shops is not randomly determined. Therefore, it is possible that confounding factors may influence whether neighbouring shops have subsidized AL and the effect of having subsidized AL may not necessarily represent the effect of competition. Interestingly, being close to a government facility, where AL is provided free of charge, did not seem to affect stocking or sales of AL. If public health facilities rarely experience shortages of AL, it would be reasonable to assume that stocking AL in nearby pharmacies would not be particularly advantageous and demand would be low. If health facilities have frequent shortages of AL, patients may leave with a prescription for AL and pharmacies near the facility may experience a high demand. Neither of these effects were measured in this study. Promoting the adoption of AL by selected outlets in various regions may be an effective way to encourage broader adoption across those regions.

Demand-side factors, such as local population density, density of fevers and measures of wealth, did not correlate with stocking and sales practice. Local fever density was marginally associated with sales of SP. Previous studies have shown a relationship between customer education, socio-economic status and purchasing ACT. More educated, wealthier individuals were more likely to purchase drugs at retail outlets with ACT, but wealthier individuals were more likely to buy SP [22]. The ability of this study to measure associations between demand-side factors and sales may be compromised by the small number of outlets falling within the population surveillance area for which demand-side variables could be assigned.

This study attempted to discern customer behaviour and preferences, for example, what attracts customers to specific outlets, from a supply-side survey. There are many important demand-side factors that were not measured. For example, consumers may be reluctant to switch from products with which they are familiar or they may prefer the one-dose regimen of SP over the six-dose, three-day treatment course of AL. Analysis of household data, including information on socio-economic indicators and treatment-seeking preferences will be useful for better understanding anti-malarial purchase decisions. In these analyses, sales volume was considered to be a dependent variable and price to be an independent predictor of sales. In reality, price is not independent of sales volume and price may increase as demand increases or *vice versa*. However, data for sales volume in just the last one-week period was used. Over such a short period, price could be expected to be relatively independent of sales volume although over longer periods, price certainly fluctuates with demand. Finally, this analysis applies to outlets that sell primarily human medicines, regardless of legal registration status, but excludes general stores that may sell anti-malarials in addition to household goods. These stores may also influence demand and sales volume in this area, but they have not been captured in the analysis.

Despite these limitations, some interesting recommendations for retail-sector antimalarial subsidies emerge from this analysis. Late or lack of adoption of subsidized AL may be a shop-level constraint. It is possible that small, rural stores with limited capital and low drug sales cannot buy new drugs until current stocks are sold. One possible remedy would be to provide an initial stock for smaller stores, either at no cost or as a loan to be repaid if the stock sells. Small stores may be risk averse and reluctant to stock newer drugs without knowing that they will be bought. Advertising and promotion of AL amongst both consumers and retailers could foster belief among shop owners that this is a product for which there will be adequate demand.

## Competing interests

The authors declare that they have no competing interests.

## Authors’ contributions

WPO conceived the study, developed the data collection, produced the analyses and the first draft of the manuscript. AO participated in study design, interpretation of results and critical revision of the manuscript. HT participated in developing the study design and analyses, and provided critical review of the manuscript. BKO participated in study design, interpretation of the results, and critical review of the manuscript. All authors read and approved the final manuscript.
